# Full-Length Enriched cDNA Libraries and ORFeome Analysis of Sugarcane Hybrid and Ancestor Genotypes

**DOI:** 10.1371/journal.pone.0107351

**Published:** 2014-09-15

**Authors:** Milton Yutaka Nishiyama, Savio Siqueira Ferreira, Pei-Zhong Tang, Scott Becker, Antje Pörtner-Taliana, Glaucia Mendes Souza

**Affiliations:** 1 Departamento de Bioquímica, Universidade de São Paulo, São Paulo, SP, Brazil; 2 ThermoFisher Scientific, Carlsbad, California, United States of America; Wuhan University, China

## Abstract

Sugarcane is a major crop used for food and bioenergy production. Modern cultivars are hybrids derived from crosses between *Saccharum officinarum* and *Saccharum spontaneum*. Hybrid cultivars combine favorable characteristics from ancestral species and contain a genome that is highly polyploid and aneuploid, containing 100–130 chromosomes. These complex genomes represent a huge challenge for molecular studies and for the development of biotechnological tools that can facilitate sugarcane improvement. Here, we describe full-length enriched cDNA libraries for *Saccharum officinarum*, *Saccharum spontaneum*, and one hybrid genotype (SP803280) and analyze the set of open reading frames (ORFs) in their genomes (i.e., their ORFeomes). We found 38,195 (19%) sugarcane-specific transcripts that did not match transcripts from other databases. Less than 1.6% of all transcripts were ancestor-specific (i.e., not expressed in SP803280). We also found 78,008 putative new sugarcane transcripts that were absent in the largest sugarcane expressed sequence tag database (SUCEST). Functional annotation showed a high frequency of protein kinases and stress-related proteins. We also detected natural antisense transcript expression, which mapped to 94% of all plant KEGG pathways; however, each genotype showed different pathways enriched in antisense transcripts. Our data appeared to cover 53.2% (17,563 genes) and 46.8% (937 transcription factors) of all sugarcane full-length genes and transcription factors, respectively. This work represents a significant advancement in defining the sugarcane ORFeome and will be useful for protein characterization, single nucleotide polymorphism and splicing variant identification, evolutionary and comparative studies, and sugarcane genome assembly and annotation.

## Introduction

Sugarcane (*Saccharum* spp.) is a C4 grass that stores large amounts of sucrose in its stems, which can account for as much as 40%–50% of the culm dry weight [1]. Sucrose from sugarcane has been used for human consumption for centuries and has recently been used for bioethanol production. Biomass from sugarcane can also be used for bioenergy production; the bagasse can be burned to generate electricity [2] and can be hydrolyzed to yield simple sugars from the complex plant cell wall, which can be fermented to produce bioethanol [3]. These features place sugarcane among the best feedstock options for future bioenergy production.

Early breeding programs used two main species for sugarcane improvement: *Saccharum officinarum* (2n = 80, basic chromosome number x = 10) and *Saccharum spontaneum* (2n = 36–128, basic chromosome number x = 8) [4]. The domesticated species, *Saccharum officinarum*, has thick and juicy culms with high sucrose content, whereas the wild species, *Saccharum spontaneum*, has thin, fibrous, low-sucrose culms and higher stress tolerance [5]. As a result of interspecies hybridization and breeding strategies, sugarcane cultivars are now highly polyploid and aneuploid, with 10–12 sets of chromosomes and a monoploid genome size of 760–930 Mb. Among the 100–130 chromosomes present in modern varieties [6], 70%–80% are derived from *Saccharum officinarum*, 10%–20% are derived from *Saccharum spontaneum*, and 10% are recombinants of these two species [7]–[9]. Recently, breeding efforts are aiming to increase energy content (GJ/ha) using *Saccharum spontaneum* genotypes to produce an Energycane, a higher yield cane, with increased fiber content and higher tolerance to drought.

The sugarcane complex genome represents a great challenge for molecular studies. Despite recent advances [10]–[16], biotechnology for sugarcane analysis is less advanced than that for other economically important crops. For example, compared with sorghum, maize, and rice, sugarcane does not have a draft genome sequence available or a well-annotated transcriptome. A multinational effort to produce a reference sugarcane genome (sugarcanegenome.org) is in progress, but many obstacles must be overcome, particularly because a high number of closely related homologs, paralogs, and alleles collapse into single contigs during the assembly.

To date, functional genomics and transcriptomics studies in sugarcane have relied primarily on expressed sequence tag (EST) and microarray datasets [17]. Despite their limitations, these studies produced important information on sugarcane gene functions [17]–[21]. Experiments addressing sucrose accumulation [22], [23], stem and cell wall development [24], [25], and responses to hormones and stresses [26], [27] have improved our understanding of mechanisms mediating sugarcane growth; however, there is still much to learn. Thus, to obtain insights into the functions of putative trait-related genes and unknown proteins, we must first obtain full-length transcripts.

In this paper, we present the development of a protocol to produce full-length cDNA libraries for cloning and next generation sequencing (NGS), using a commercial hybrid (SP803280) and two ancestor genotypes (*Saccharum officinarum* and *Saccharum spontaneum*). We also present high-throughput sequencing data from the set of open reading frames (ORFs) in the sugarcane genome (i.e., the sugarcane ORFeome). Defining this full-length cDNA dataset will be critical for large-scale protein characterization and genome assembly and annotation, facilitating sugarcane improvement. The use of the two ancestor genotypes will help define genes and regulatory networks that may underlie the distinct phenotypes that can be useful for improving sugarcane yield and for designing new varieties for bioethanol generation.

## Materials and Methods

### Plant materials

Leaves, immature and intermediate internodes samples from field-grown SP803280 plants were sampled after plants were cultivated for 9 months. The immature internode was considered the first internode at the apex of the culm. The intermediate internode was considered the fifth internode from the immature internode. *Saccharum officinarum* (Caiana listrada) and *Saccharum spontaneum* (IN8458) were grown in a greenhouse, and leaf samples were harvested after plants were cultivated for 11 months. Each sample was a pool from five individual plants. Field samples were cultivated at Centro de Ciências Agrárias - Universidade Federal de São Carlos, city of Araras, state of São Paulo, Brazil (http://www.cca.ufscar.br/). GPS coordinates: 22°18′46.4″S 47°23′09.6″W. No specific permissions were required for these locations/activities and field studies did not involve endangered or protected species.

### RNA isolation, full-length first-strand cDNA (FLFS cDNA) synthesis, and enrichment

Total RNA was isolated using TRIzol (Life Technologies) followed by polyA mRNA isolation using a FastTrack MG mRNA Isolation kit (Life Technologies). The polyA mRNA (10 µg) from each sample was used for first-strand cDNA synthesis using SuperScript III (Life Technologies) and oligo-dT_21_VN primers (1 µg) for direct sequencing or an attB2-dT_19_VN primer (GGGGACAACTTTGTACAAGAAAGTTGGGT (T)_19_VN) for library construction. Reactions were carried out in 1× reaction buffer and incubated at 45°C for 40 min, 50°C for 40 min, and 55°C for 40 min. To enrich full-length cDNA, the mRNA:cDNA hybrid sample was treated with RNAse One (Ambion) in the same buffer and incubated at 37°C for 30 min. The sample was extracted using phenol:chloroform:isoamyl alcohol (Life Technologies) and precipitated by ethanol. The cDNA:mRNA sample was resuspended in 600 µL of 1× TE buffer containing 100 mM NaCl_2_, followed by addition of 3 mg Cap antibody-conjugated magnetic beads (160 µg antibody/mg beads, Life Technologies) and gentle rotation at room temperature for 1 h. The beads were washed three times with 1× TE buffer containing 100 mM NaCl_2_. For library construction and 454 sequencing (Roche), the full-length enriched cDNA:mRNA sample was eluted from beads in 300 µL buffer containing 1.5 M guanidine isothiocyanate, 20 mMTris-HCl (pH 7.5), and 10 mM EDTA and incubated at room temperature for 1 h. For direct sequencing, the full-length enriched single-stranded cDNA was eluted from cDNA:mRNA beads in 1× RNase H buffer containing 2 units of RNAse H (Life Technologies) and 12 µg of RNAse A (Life Technologies) for 30 min at 37°C, followed by incubation at 95–100°C for 5 min. The eluted FLFS cDNA was precipitated by ethanol and used for subsequent experiments.

### cDNA library construction and cloning

The full-length enriched cDNA:RNA was used for second-strand cDNA synthesis in a 150-µL reaction containing 1× second-strand cDNA synthesis buffer (Life Technologies), 1.3 mM DTT, 266 µM dNTP, 10 units *E.coli* DNA ligase, 40 units *E.coli* DNA polymerase, and 2 units *E.coli* RNAse H. The samples were incubated at 16°C for 2 h, and the double-stranded cDNA was polished by incubation with T4 DNA polymerase at 16°C for 5 min. A double-stranded 5′ adaptor containing an attB1 site (top strand: TCGTCGGGGACAACTTTGTACAAAAAAGTTGGA; bottom strand: PHO-TCCAACTTTTTTGTACAAAGTTGTCCCC) was ligated to the double-stranded cDNA. The cDNA sample containing an attB1 site at the 5′end and an attB2 site at the 3′end was cloned into the pDNOR222 vector (Gateway, Life Technologies) in a 20-µL recombination reaction containing 1× TE buffer and 4 µL BP enzyme mix (Life Technologies) and incubated at room temperature overnight.

### 454 Titanium sequencing

Double-stranded cDNA was fragmented using the Covaris S220 system (Covaris, Woburn, MA, USA) to target DNA fragments ranging in size from 400 to 1000 bp following the manufacturer's protocol using MID tags for each sample.

### Ion PGM sequencing

To maintain transcriptome complexity, we used 75% FLFS cDNA and 25% original polyA mRNA from the sample preparation for Ion PGM sequencing (Life Technologies). The FLFS cDNA sample was fragmented using the Covaris S220 system (Covaris) to target DNA fragments ranging from 300 to 500 bp, following the manufacturer's protocol. A DNA Ion-adaptor mixture (containing the standard Ion A and P1 sequences) was ligated to the FLFS cDNA fragments. For the polyA mRNA, RNAse III was used for fragmentation, followed by size selection (300–500 bp), adaptor ligation, and reverse transcription using the “Ion-Total RNA-Seq” protocol (version 2) for preparation of 300-base-read RNA libraries (Life Technologies). The adapted cDNA fragments derived from FLFS cDNA and mRNA were equally mixed for emulsion PCR using the One Touch 2 system following the Ion PGM Template OT2 400 base protocol (Life Technologies). The enriched template-positive Ion sphere particles were loaded onto the Ion 318 chips following manufacturer's protocol. The raw data were trimmed, and FASTQ files were generated using Ion plug-in software.

### Raw reads processing

Data from 454 and Ion PGM were processed and filtered individually. The Ion PGM raw data were trimmed in Torrent Suite (version 3.6.2) to remove primer, adaptor, and low-quality base calling sequences. The FASTQ files containing Q20 reads were generated using Ion plug-in FastqCreator software (3.6.0). Reads from the 454 platform were processed and trimmed with Prinseq [28]. First, we applied a quality filter by trimming reads using mean Phred quality scores below 20, reads with at least 5 N's or 5 poly-A/T's in the ends, and removing reads with minimum length of 70 after trimming. Next, we applied the ‘dust’ method to remove low complexity sequences. All reads were deposited in the Sequence Read Archive (SRA-NCBI) database under accession number SRP042605.

### *De novo* transcriptome assembly

Ion PGM and 454 processed reads were combined and assembled using Trinity 2013-02-25 software [29]. Reads from all five samples were combined, and the minimum sequence length in the assembly was set to 200 bp. Trinity software processed data sequentially through three modules, which allows for recovery of transcript isoforms using the de Bruijn graph algorithm [30].

### Gene prediction

We used three ESTScan scripts to create a sugarcane codon-usage matrix and to predict ORFs: (i) *extract_mrna*, using 832 manually annotated genes from 303 Sugarcane BAC sequences [31] with a mean size of 1,820 bp, composed of at least two exons and 100 bp in 5′ and 3′ untranslated regions (UTRs); (ii) *prepare_data*, to separate genes in the training and test groups according to GC-content, to mask redundancies, and to classify coding and noncoding parts of genes; and (iii) *build_model*, to create the matrix using the previous data. This matrix was used for protein prediction with ESTScan [32].

### Functional annotation of sugarcane transcripts

All contigs were used for comparative and functional genomic analyses. Putative genes with functional annotations were compared in the following order, for species with more specific transcript hits (Table S1), against each grass genome dataset: *Sorghum bicolor*, *Oryza sativa*, *Zea mays*, *Brachypodium distachyon*, *Panicum virgatum*, and *Setaria italica* (Phytozome v9.0). Comparisons were performed by BLAST search (e-value 1e-05) and were processed with an *in-house* script based on two methods: single-directional best hit (SBH) information (blastx) and bidirectional best hit information, in both forward (BLASTX) and reverse directions (TBLASTN), taking each gene in genome A as a query compared against all genes in genome B, and vice versa [33]. The mapping step allowed the cross-species annotation (i.e. to take the annotation from a grass gene and assign it to the matching sugarcane contig), for BLAST hits with bit scores higher than 30, following the above species order. Since the bit scores of a gene pair a and b from two genomes A and B, respectively, can be different in forward and reverse orientations, and because the top scores do not necessarily reflect the order of the rigorous Smith-Waterman scores, we used the bidirectional hit rate (BHR) and selected genes with BHRs greater than 0.95 [33]. To annotate the assembled contigs according to GO terms [34], we used the BHR method as described above to determine gene ontologies. All predicted proteins were categorized according to PFAM domains [35] using HMMPFAM from InterProScan software [36] with an e-value threshold of 1e-03.

Next, we identified sugarcane metabolic pathways for predicted proteins using the KEGG Automatic Annotation Server (www.genome.jp/kegg/kaas) with the eukaryote and plants dataset, which assigns KO identifiers as controlled vocabularies to sets of new sequences based on BHRs ≥30, excluding non-plant pathways.

### Functional activity scores

Following the functional class scoring (FCS) approach [37], we developed an algorithm that integrated transcript expression profiles and metabolic pathways to estimate the activities of metabolic pathways using *in-house* scripts and R-statistics modules. To define and compare the functional activities of NATs for the metabolic pathways in each sample, we initially selected the significantly expressed antisense transcripts and then estimated the pathway activity based on the antisense transcripts identified in each pathway and their relative abundances from each sample. The pathway activity comparisons represented the likelihood that a metabolic pathway was active and the degree of activation. To calculate the relative pathway activity (PA), the pathway(s) associated with each antisense expression profile was retrieved. Then, we summed the gene intensities for each pathway to obtain the total pathway score and normalized it dividing by the proportion of detected antisense transcripts for its respective pathway. The score for PA was calculated as:
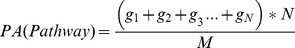
where: *g_j_* is the intensity of gene *j* identified from pathway P, *N* is the total number of genes detected in pathway P, and *M* is the total number of genes part of pathway P.

Then, the scores were log2-transformed and centered by the median value. Subsequently, we performed a hierarchical clustering using the average method (for pathways) and the Spearman correlation method (for samples). The cut in the tree to decide the number of clusters in the hierarchical clustering was determined using the R package NbClust [38] with the “Euclidean distance” to create the dissimilarity matrix.

### Reads mapping and identification of antisense transcripts

The six grass genomes cited above, SUCEST transcripts [39], and Trinity assembled contigs were used to map reads from each cDNA library to identify the expression levels of contigs in each genotype or sample and the presence of antisense transcripts using the Bowtie aligner [40] with the following parameters: *very-sensitive-local: (-D 20 -R 3 -N 0 -L 20 -i S,1,0.50); p: 32* (number of threads); and hits: ≥100 bp (minimum alignment length). Only reads from Ion PGM sequencing were used for antisense identification since the protocol used allowed to kept the strand orientation, what was not possible for 454 sequencing.

### TFs

TF families were obtained from predicted proteins based on specific rules as established in GRASSIUS [41]. The domain identification involved a HMMPFAM search from InterProScan against all available PFAM hidden Markov models, keeping only significant hits with an e-value threshold of 0.001. After that, we applied the script with the GRASSIUS rules for the identification and classification of putative TFs.

### Prediction of full-length transcripts

To estimate the number of full-length sugarcane transcripts, we used the full-length CDS genes from six grasses as references: *Sorghum bicolor*, *Zea mays*, *Panicum virgatum*, *Setaria italica*, *Oryza sativa*, and *Brachypodium distachyon*. These genes contained the complete mRNA (5′UTR+CDS+3′UTR) (Table S2). Identification of full-length sugarcane transcripts was conducted in three analyses. The first two were based on the alignment between sugarcane contigs and CDSs of full-length species genes by BLASTN, with parameters as follows: e-value threshold: 1e-15, *max_target_seqs: 1, outfmt: 6, dust: NO, num_threads: 20*, only contigs with coverage ≥50%, respective gene hit coverage ≥95%, and identity ≥80%. In Analysis I, we considered transcripts to be full-length only if they completely covered the first gene hit, according to the above criteria, and such genes and contigs were disregarded in Analysis II. In Analysis II, we selected full-length genes of each species if two or more contigs covered the given gene, based on the above criteria. In both analysis, the genes were analyzed in a specific species order (*Sorghum bicolor*, *Zea mays*, *Panicum virgatum*, *Setaria italica*, *Oryza sativa*, and *Brachypodium distachyon*), based on the species with more specific transcript hits (Table S1). Transcripts identified as full-length in comparison to *Sorghum bicolor* genes were not employed in the analysis in *Zea mays*, and those identified as full-length in comparison to *Zea mays* were not employed in the analysis in *Panicum virgatum* and so on. In Analysis III, we calculated the mean CDS size (1,030.85 bp, Table S2) for full-length genes from the six grasses, and contigs not classified in the two previous analyses were selected as putative full-length genes if their CDS sizes were greater than 1,030.85 bp and complete CDS (including the start and stop codons) similar to [42]. Prediction of paralogs and gene families (see below) allowed the identification of uniquely represented full-length contigs by clustering alleles, variants and paralogs of a given gene. Full-length TFs were identified by integration between full-length transcript analysis and the TF identification as described above.

### Gene family analysis

Paralogs and orthologs were identified from the sugarcane, *Sorghum*, maize, *Oryza*, *Setaria*, *Brachypodium*, and *Panicum* putative full-length predicted proteins (Table S2) with Hieranoid software (Schreiber *et al.* 2013) by inferring pair-wise homology relationships between the translated transcripts and combining them into groups of orthologous sequences, with specific parameters (bootstrap: 1, matrix: BLOSUM62, *group_overlap_cutoff*: 0.5).

## Results and Discussion

### Library construction and cDNA sequencing

**Figure 1:**
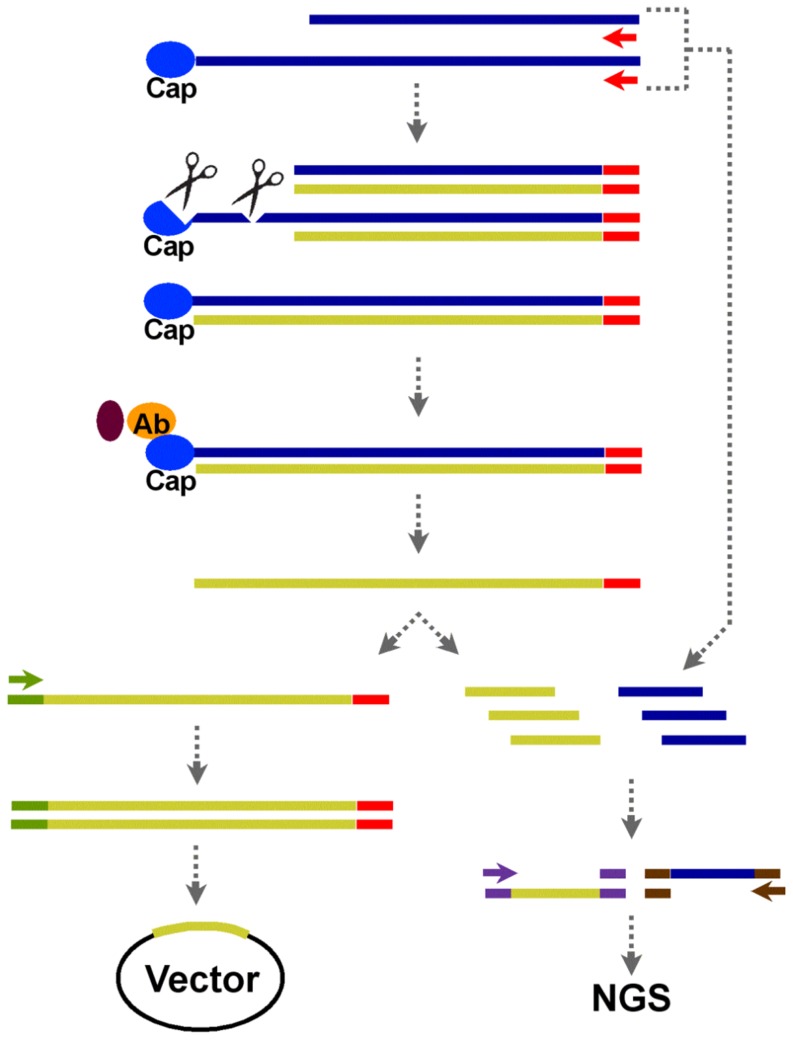
Full-length enrichment for library cloning and next generation sequencing (NGS). Full-length (blue line with 5′ cap) or truncated (short blue line without 5′ cap) mRNAs were reverse transcribed into first-strand cDNA using oligo-dT primers (red arrow). The mRNA:cDNA hybrid was treated with RNase I (scissor) to remove the single-stranded RNA that was not fully extended by the first-strand cDNA, followed by selection for full-length transcripts using Cap-antibody magnetic beads to enrich the full-length mRNA:cDNA. The full-length single-stranded DNA (FLssDNA) was eluted from beads and used for both cDNA library cloning (lower left) and NGS (lower right). For full-length library cloning, a double-stranded adaptor (green) was linked to the 5′ end of ssDNA. Second-strand cDNA synthesis was then carried out, followed by cloning into a vector. For NGS, the full-length enriched ssDNA was fragmented by sonication to target fragments in the range of 200–400 bp, followed by ligation of the double-stranded DNA sequencing adaptor mixture (purple) to 3′ and 5′ ends of ssDNA. To maintain the complexity of the library while enriching the full-length cDNA for NGS, the original polyA mRNA was also fragmented using RNAse III, followed by ligation of the double-stranded RNA sequencing adaptor mixture (brown) to 3′ and 5′ ends of mRNA. After first- and second-strand synthesis, the polyA and capped mRNA and polyA and non-capped mRNA samples were mixed in a 3∶1 ratio and applied to the downstream NGS procedure.

The genotypes *Saccharum officinarum* (Caiana listrada) and *Saccharum spontaneum* (IN8458) were chosen since they are representatives of the genotypes used in early breeding programs to develop sugarcane hybrids. Variety SP803280 was chosen because it represents a common sugarcane variety model used in studies in all areas and is currently being sequenced by the sugarcane genome project (sugarcanegenome.org). Five samples were used to produce individual cDNA libraries, three from SP803280 (leaves, immature and intermediate internodes) and one from each of the ancestor genotypes (leaves only). Using these samples, we implemented a protocol for construction of full-length enriched cDNA libraries for cloning and NGS (Figure 1). Briefly, oligo(dT) primers were used to synthesize cDNA and were then treated with RNAse I to digest single-stranded RNAs (non-reverse-transcribed into cDNA) and by full-length cDNA selection using Cap-antibody magnetic beads. A fraction of each cDNA sample was ligated to barcoded double-stranded DNA adaptors for cloning. The remaining fraction was fragmented, ligated to double-stranded adaptors, mixed with original polyA mRNA that was also reverse transcribed into cDNA, and sequenced by NGS.

For cloning, all five barcoded libraries were mixed, cloned into Gateway vectors (pDNOR222, Life Technologies), and divided into two fractions based on their average insert size, i.e., approximately 1.1 and 1.4 kb (Table 1). Thus, the sugarcane cDNA library was consistent with previously reported full-length libraries for other plants. The cDNA average insert lengths of 1.1 and 1.4 kb were similar to those of tomato (1,418 bp) [43], *Arabidopsis* (1,445 bp) [44], and poplar (1,045 bp) [45] and longer than that of *Brachypodium* (808 bp) [46]. Twenty-six randomly selected clones were sequenced by the Sanger method from both cDNA ends, achieving full-length inserts from 90% of clones after excluding low-quality sequences and repeated clones (Table 1).

**Table 1:** Description of cloned full-length cDNA libraries.

Library fraction	Number of clones	Mean length (kb)	Clones sequenced	Clones analyzed	Sugarcane specific	Full-length	% of putative FL
L	3×10^8^	1.4	13	8^a^	0	8	90% (19/21)^b^
S	7.5×10^8^	1.1	13	13	1	11	

a Excluding low-quality sequences and repeated clones.

b Excluding sugarcane-specific transcripts.

The libraries were sequenced by two different NGS platforms. Eight runs were carried out (five on an Ion PGM instrument [Life Technologies, Carlsbad, CA, USA] and three on a 454 Titanium instrument [Roche, Branford, CT, USA]), totaling 31,663,934 trimmed reads and 6.6 Gb of sequence (Table 2). All trimmed reads were assembled with Trinity software [29], yielding 195,765 contigs with a mean contig size of 684 bp and N50 of 963 bp (Table 2). Around 20% of contigs were 1 kb or more in length (Table 2).

**Table 2:** Data from Ion PGM and 454 sequencing (after trimming) and Trinity assembly output.

Description	Ion PGM	454
Runs	5	3
Number of reads	29,260,184	2,403,750
Yield (Gb)	5.69	0.97
Mean read length (bases)	194	404
Median read length (bases)	197	436
Longest read (bases)	645	861
Shortest read (bases)	8	50
**Trinity Assembly**		
Number of contigs	195,765	
Total bases in contigs	133,946,191	
Longest contig (bp)	8,854	
Shortest contig (bp)	201	
Number of contigs >1 Kb	39,266 (20.1%)	
Mean contig length (bp)	684	
Median contig length (bp)	462	
N50 contig length (bp)	963	

A recent work by Cardoso-Silva and colleagues reported a *de novo* assembly of the sugarcane transcriptome using GAIIx Illumina 2×36 bp (paired ends) of six sugarcane commercial varieties from three different breeding programs [47], which included the genotype SP803280 used in this present work. However, the experiments carried out in this previous analysis [47] did not focus on full-length enrichment as described here. Instead, they aimed to identify molecular markers in the transcriptome that could be correlated to different phenotypes among the six varieties sampled, particularly resistance to rust and sucrose accumulation. Besides the GAIIx Illumina platform gives shorter reads than PGM and 454, it fits better to the expression profile analysis and the study of molecular markers, such as SSRs and SNPs. The authors produced over 445 million reads in six runs, which yielded almost five times more sequence (32 Gb) than reported here (6.6 Gb, Table 2). They reported 119,768 assembled contigs (72,269 unigenes) with an average length of 921 bp, N50 1,367 bp, and 18,624 unigenes with 1 kb or more in length. The results regarding average length and N50 are higher than those of our results (see Table 2). However, the work by Cardoso-Silva and colleagues excluded all contigs shorter than 300 bp, which certainly helped to increase these two values. If we assumed the same criteria used by Cardoso-Silva (contig minimum length  = 300 bp and excluding isoforms), the N50 increased from 963 bp (Table 2) to 1,071 (data not shown). These results suggest that sequencing depth may be more important than read length to produce assemblies with longer transcripts on average, since better results of average length and N50 were achieved by shorter reads from the GAIIx platform [47] than from similar technologies and longer reads from the 454 platform (this present study). However, recent publications have shown that longer reads could be useful for the identification of splicing isoforms, determination of the exon-intron structure, allele discrimination, and single nucleotide variations [48], [49], as in the case of Iso-Seq (Pacific Bioscience, Menlo Park, CA, USA), which generates longer reads than the 454 platform, using amplification-free long-read sequencing, originated from a single RNA molecule. Despite the higher error rate of the Iso-seq method compared to other technologies, these very long reads and their circular consensus reads could be used as a scaffold for *de novo* assembling, and the error could be corrected by short reads from other platforms [50].

### Comparisons with other species and among genotypes revealed a high number of sugarcane-specific transcripts, but a low number of genotype-specific transcripts

**Figure 2:**
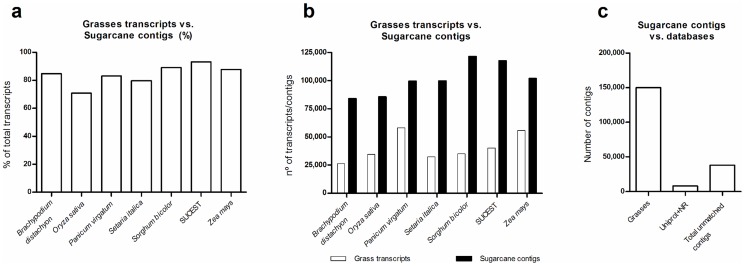
Number of grass transcripts mapping to sugarcane contigs. A, Percentage of total grass transcripts mapping to sugarcane contigs. B, Total grass transcripts mapping to sugarcane contigs (white bars) and total sugarcane contigs mapping to each grass database (black bars). C, Total sugarcane contigs mapping to grasses, Uniprot, and NR databases and total unmatched sugarcane contigs (putative sugarcane-specific transcripts).

**Figure 3:**
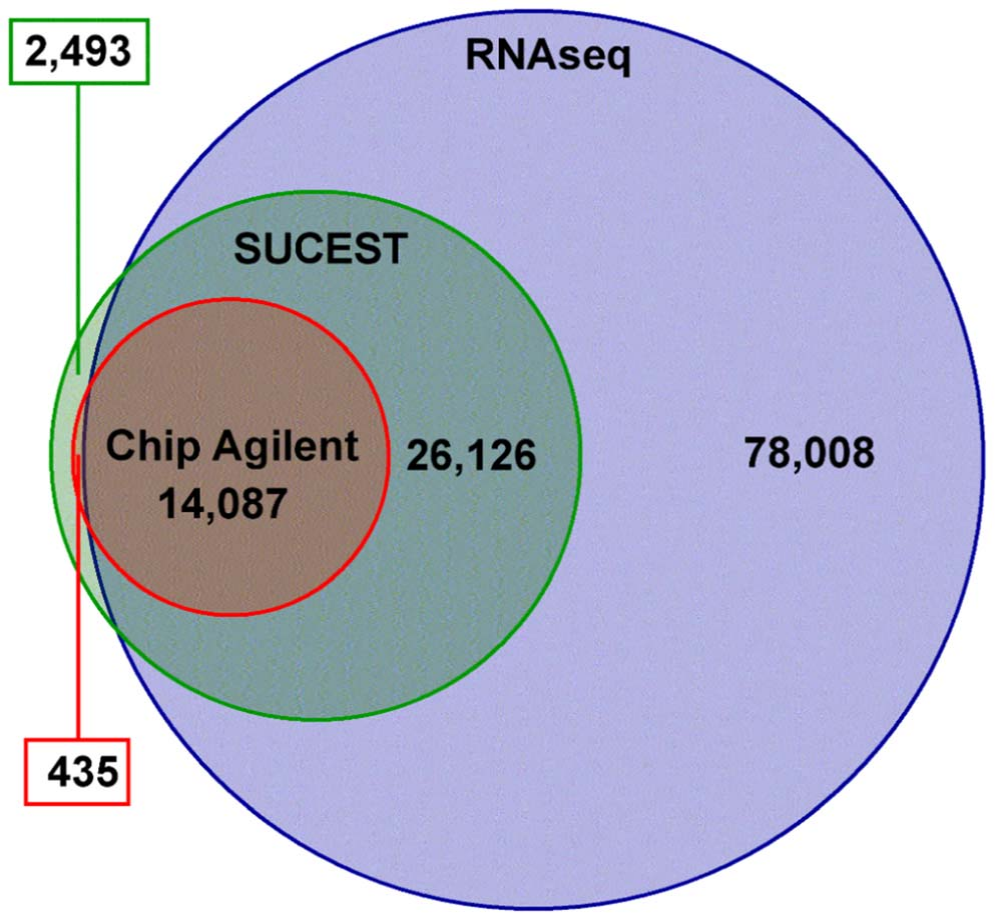
Venn diagram comparing sugarcane transcripts as obtained by RNAseq (blue, this work), SUCEST (green) [51], and those that have been studied using oligoarrays in customized Agilent sugarcane chip (red) [26], [64]. Green and red boxes show the number of transcripts present in the SUCEST data (green) and the Agilent Chip (red) but not in the sugarcane ORFeome (this work).

Sugarcane contigs were mapped against the predicted genes of six grasses plus the main sugarcane EST database, SUCEST [39], [51] (http://sucest-fun.org). At least 70% of all grass genes showed a matching contig, with higher percentages for closely related species, such as sorghum (Figure 2A and 2B). About 7% of SUCEST sequences did not match any contig (Figure 2A); this may be explained by tissue-specific genes that are present in SUCEST but were not sampled in this study, such as those found in roots, lateral buds, and flowers [51]. Interestingly, 78,008 contigs did not match any SUCEST sequence (Figure 3), and only 3,086 contigs were specific to *Saccharum officinarum and/or Saccharum spontaneum* (Table S3); since only 15% of SUCEST reads were generated from leaf and internode tissues [51], these may be novel and specific sugarcane transcripts in leaf and internode tissues. This represented an increase of 1.8-fold compared to the SUCEST database, which contains around 43,000 sequences. Moreover, 38,195 contigs (19.5%) may represent sugarcane-specific transcripts since they did not match any analyzed public database (Figure 2C). This percentage was similar to that in SUCEST (20%–25%) [52], but much higher than reported for the tomato full-length library [43], in which around 6% of all sequenced transcripts have no match.

**Figure 4:**
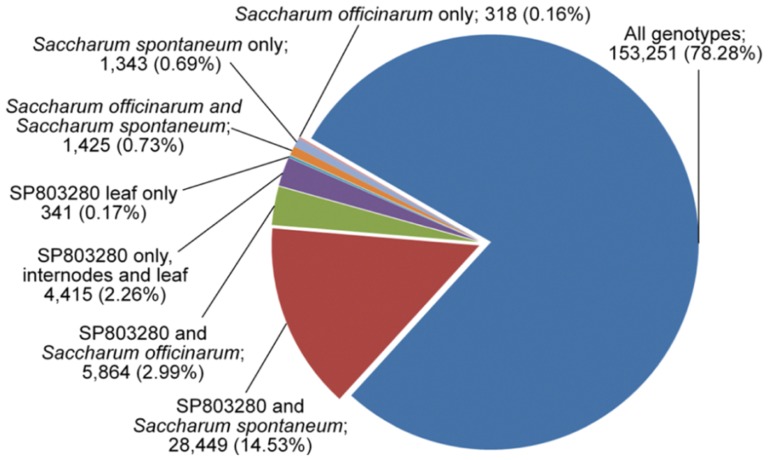
Number of contigs expressed by genotype.

After mapping all reads from each sample against the assembly, we identified only a few genotype-specific contigs, and less than 1.6% were not present in SP803280, i.e., were expressed only in *Saccharum officinarum* (0.16%), *Saccharum spontaneum* (0.69%), and both ancestors (0.73%), while 153,251 (78.28%) contigs were expressed in all three genotypes (Figure 4 and Table S1). A note should be made here that tissues were sampled in parallel under similar conditions from green house grown plants in the case of ancestor genotypes and field-grown plants in the case of SP803280, therefore expression profiles should be noted with care. The divergence between *Saccharum officinarum* and *Saccharum spontaneum* took place 1.5–2 million years ago [53], and this low number of genotype-specific transcripts (contigs) suggests that few major changes occurred in expressed regions of the genome. The genetic differences that account for major phenotypic differences (e.g., sucrose accumulation, yield, and stress tolerance) may be related to differentially expressed genes and alleles, single-nucleotide polymorphisms (SNPs), splicing variants, and so on. This hypothesis is in agreement with recent work on the sucrose synthase gene family in ancestor genotypes [54], which exhibits significant differences in SNP frequency and resulting minor alterations in protein sequences. In addition, polymorphisms are more frequent in *Saccharum spontaneum* than in *Saccharum officinarum* and hybrid genotypes [55]. Compared to sorghum, which diverged from sugarcane 8–9 million years ago [53], we observed greater differences in the presence/absence of transcripts; 121,562 contigs matched sorghum genes (Figure 2B), indicating that 37.9% of sugarcane contigs differed from sorghum transcripts. Further analysis on these results will improve our understanding of genome evolution and grass species divergence.

BLASTN comparisons between the two sugarcane *de novo* assemblies described in this present work (195,765 contigs) and by a previous study [47] (72,269 unigenes) revealed 15% and 23% of contigs and unigenes, respectively, that did not match (data not shown). This high number of unmatched transcripts between these two assemblies suggests that sugarcane might present a very complex transcriptome, with possible variety-specific transcripts. However, the environmental conditions and tissues harvested were not the same in these two studies, and therefore, sampling conditions may partly explain these differences.

### Functional annotation of full-length sugarcane cDNA libraries

Functional annotation was carried out for sugarcane contigs based on Gene Ontology (GO) [34], Kyoto Encyclopedia of Genes and Genomes (KEGG) [56], PFAM [35], and Phytozome (v9.0) (www.phytozome.net) databases (Table 3). We found 78,273 (40%) contigs with categories in GO terms. The three main categories, i.e., “cellular component”, “molecular function”, and “biological process”, showed “nucleus”, “protein binding”, and “regulation of transcription, DNA dependent”, respectively, as the most frequent categories (Table S4). Compared to the sugarcane transcriptome annotation by Cardoso-Silva [47] (14,983 unigenes annotated in GO terms), a higher number of transcripts was annotated by the GO database, even when using the same criteria as used in the previous study [47] (i.e., minimum contig length and excluding isoforms; 24,085 transcripts, data not shown). KEGG pathway annotation yielded 14,298 contigs assigned into 132 pathways; 7,385 and 2,761 of these contigs were assigned into “metabolic pathways” and “biosynthesis of secondary metabolites”, respectively (Table S4). Using ESTScan [32] we found protein prediction for 187,935 (96%) contigs (Table 4) and these proteins showed a mean length of 188 amino acids. These predicted protein sequences were compared with PFAM domain database, obtaining 57,526 matching proteins, with protein kinase (Pkinase) as the most frequent domain (Table S4). Next, using the results from mapping sugarcane contigs against grasses (Figure 2), categories from grass best hits were retrieved and assigned to the respective matching sugarcane contig, resulting in 139,980 contigs with 6,274 different categories (Table 3). “Unknown”, “NB-ARC domain”, and “protein kinase superfamily” were the three most frequent categories (Table S4). Interestingly, although the plants were not noticeably stressed at the time of sampling, we identified enriched stress-related categories, such as “response to salt stress” and “defense response” in GO and NB-ARC domain [57] in Phytozome and PFAM databases (Table S4).

**Table 3:** Functional annotation of sugarcane full-length cDNA contigs.

Database	Number of annotated contigs (% from total)	Number of different annotations/categories^a^
GO	78,273 (40%)	5,876
KEGG pathways	14,298 (7.3%)	132
PFAM	57,527 (29.4%)	3,822
Phytozome^b^	139,980 (71.5%)	6,274

a For some databases, several contigs showed more than one annotation or categorization.

b Based on annotation from *Sorghum bicolor, Z. mays*, *P. virgatum*, *Setaria italica*, *O. sativa*, and *B. distachyon*.

**Table 4:** Protein prediction of sugarcane contigs.

Description	Number
Contigs	195,765
Contigs with protein prediction	187,935
Contigs with no prediction	7,830
Mean length (aa)	188
Median (aa)	116
Longest predicted protein (aa)	2,952

aa =  amino acids.

**Figure 5:**
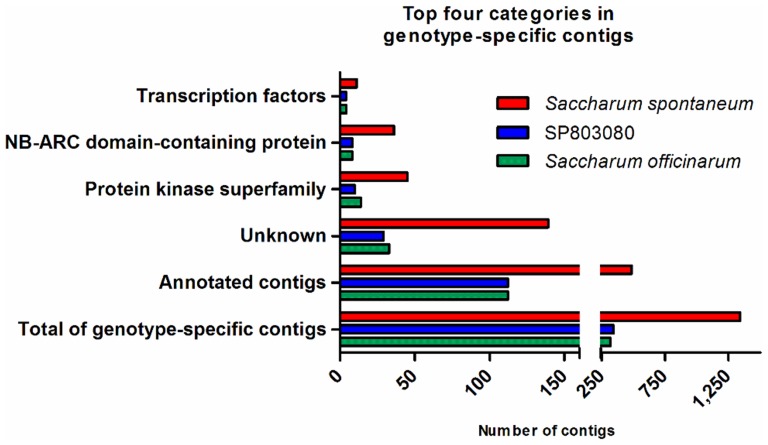
Top four categories of genotype-specific contigs based on Phytozome annotations. Only contigs from leaf samples in each genotype were considered.

We selected the categories of genotype-specific contigs (Table S5), focusing in contigs expressed only in leaves, to identify differences among genotypes. Figure 5 shows the four most frequent categories, which were the same for all three genotypes. *Saccharum spontaneum* is the genotype responsible for introducing stress tolerance into hybrid sugarcane [5]. The presence of several individual contigs containing the stress-related NB-ARC domain [57] among genotype-specific transcripts, especially in *Saccharum spontaneum* (36 contigs), suggested their involvement in this phenotypic difference. Moreover, the presence of a high number of kinases and transcription factors reinforced the importance of signal transduction for phenotypic differences among *Saccharum* genotypes. All of these genotype-specific transcription factors were actually small contigs, ranging from 200 to 450 bp (data not shown), and were therefore unlikely to be complete genes. Instead, we consider that these may represent different variants of the same gene.

### Natural antisense transcripts (NATs) appeared to affect several biological processes

**Figure 6:**
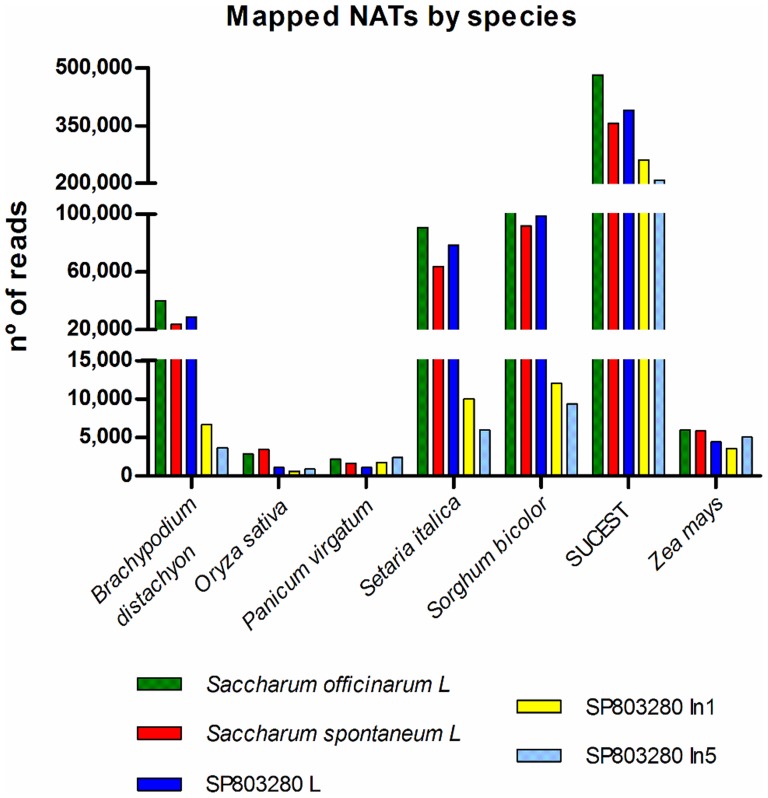
Number of reads identified as natural antisense transcripts (NATs), based on their orientation on alignment to grass genes. Tissues are indicated as follows: In1, immature internodes; In5, intermediate internodes; L, leaves.

Another important feature in sugarcane is the presence of NATs. NATs modulate the expression of their sense counterparts through several mechanisms [58]. NAT expression has been studied in several plants, such as maize [59], rice [60], *Arabidopsis* [61], [62], and *Brassica rapa* [63]. Studies have also suggested that they may be important for regulating gene expression under stress conditions [26], [63]. In sugarcane, NAT expression has been found to be responsive to drought [26] and is modulated in a circadian manner [64]. Since sugarcane does not have a draft genome with predicted gene models, antisense identification was performed here by mapping the reads against gene models of other grasses using only Ion PGM reads as it is the only protocol used that maintained strand orientation. We detected NATs in all samples (Figure 6); their expression was higher in leaf samples, which was particularly evident when the analysis was carried out in *Brachypodium distachyon*, *Setaria italica*, *Sorghum bicolor*, and SUCEST (Figure 6). These data suggested that some processes in leaves might be more dependent on or affected by antisense mechanisms than in internodes. However, this is different from data reported for *Arabidopsis*, where different tissues (roots, leaves, and inflorescence) have comparable expression levels of NATs [62]. Furthermore, we identified no significant differences in total NAT expression among ancestral genotypes and the commercial hybrid (Figure 6), suggesting that this feature was conserved among *Saccharum* species, regardless of domestication and genome hybridization. Using SUCEST as reference, adding up all antisense reads in all five libraries, there are close to 1,700,000 antisense reads, which means ∼5.8% of antisense reads (1,700,000/29,260,184).

**Figure 7:**
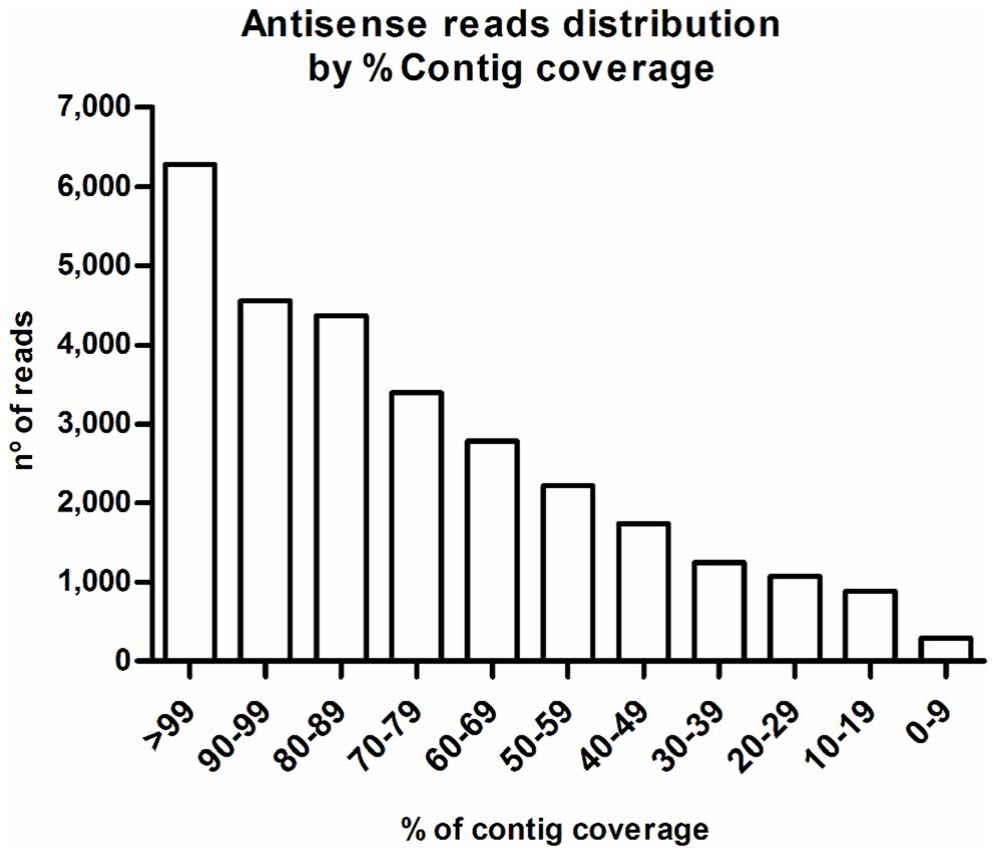
Number of contigs and percentage of coverage by antisense reads. Non-overlapping contig coverage by antisense reads was calculated for all contigs showing antisense expression (28,844 contigs).

Recently, NATs were predicted to represent approximately 70% of all *Arabidopsis* protein-coding loci, suggesting that NATs are much more widespread than previously thought [62]. Here, antisense mapping yielded 28,884 contigs (14.7%) with antisense reads (Figure 7), a much lower percentage than estimated for *Arabidopsis*. To obtain insights into the roles of these NATs, contigs showing NAT expression were assigned into biological processes by mapping them against the KEGG pathway database to cluster different proteins or enzymes of a given pathway into a single “expression” value for this KEGG pathway (Figure S1). A wide range of pathways is represented, with 94% (126/134) of plant KEGG pathways showing antisense expression, suggesting that NATs were widely present in the sugarcane transcriptome. Leaf-specific pathways, such as “carbon fixation” and “photosynthesis”, showed NAT expression (Figure S1), correlating with the higher number of NATs in leaves (Figure 6). Pathways with higher “expression” of NATs were different in each genotype. *Saccharum spontaneum* showed “alanine, aspartate, and glutamate metabolism” as NAT-enriched pathway, whereas SP803280 and *Saccharum officinarum* showed “indole alkaloid biosynthesis” and “lipoic acid metabolism”, respectively.

### A dataset of 17,563 unique sugarcane full-length genes

**Figure 8:**
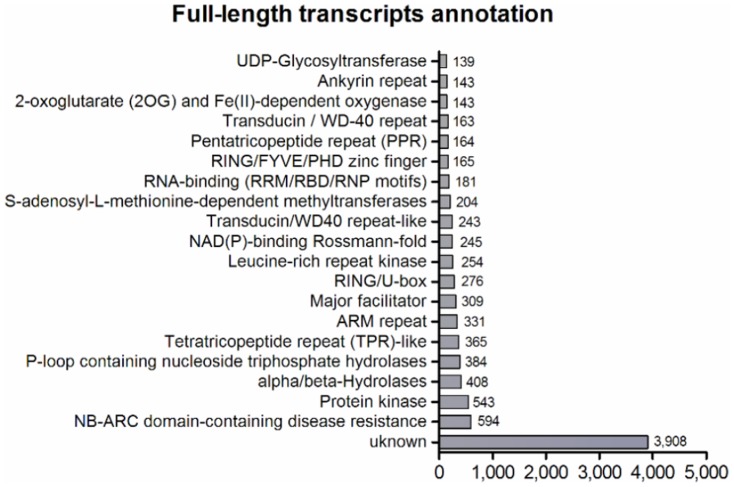
Functional annotation of full-length contigs (40,407). The graph shows the 20 most frequent categories from Phytozome annotation. In total, 5,038 categories were observed (Table S4).

We conducted a three-step analysis to estimate the number of full-length transcripts in our ORFeome. Analysis I (Table 5) considered a contig as full-length if a single contig covered more than 95% of a grass CDS with an identity of ≥80%, yielding 9,960 (9,410 unique) sugarcane full-length contigs. After manual analysis of alignment results, we observed that several full-length grass genes were covered by two or more contigs (Figure S2). These contigs represent a single transcript that was not assembled together, probably due to the high stringency of the assembler, problems in *de novo* assembly, or lack of sequencing depth. Therefore, we carried out a second analysis (Analysis II, Table 5) using the same criteria of coverage and identity cited above, but considering the non-overlapping coverage of different contigs in a given gene. This analysis did not take into account the contigs identified in Analysis I and resulted in 26,384 contigs representing 4,027 genes (3,952 unique genes), with an average of 6.55 contigs per full-length gene. Finally, using the remaining contigs, Analysis III searched for contigs with a complete predicted ORF (start and stop codons) and took into account the average CDS length of full-length grass genes (Table S2), comparing it with the length of the predicted ORF of the sugarcane contig. If the CDS size was bigger than the average CDS length, the contig was considered full-length; 4,243 contigs fit these criteria. As a result of these three analyses, we identified 40,587 contigs representing 17,563 unique, full-length genes (Table 5). Assuming 33,000 as the approximate total number of genes in sugarcane [41], we identified 53.2% of all sugarcane full-length sequences. This number of unique full-length transcripts is much higher than reported for other plant full-length libraries, such as poplar (3,990) [45], tomato (11,502) [43], and *Brachypodium* (10,513) [46], even though the estimated number of total genes in the genome is similar among these plants (34,000 for tomato, 31,000 for *Brachypodium*, and 33,000 for sugarcane). Notably, identification of full-length sugarcane genes was carried out by *de novo* assembly in this study, in contrast to the full-length libraries cited above, which used an available draft genome to facilitate the assembly. Among all 40,587 contigs identified in the full-length analysis (Table 5), 40,407 showed a match against the Phytozome database (Table S4). Unknown proteins, NB-ARC domains, and protein kinases were the most frequent categories (Figure 8), similar to the results for all annotated contigs.

**Table 5:** Number of full-length transcripts identified by each analysis.

	Analysis I		Analysis II			Analysis III
	First hit against CDS		All hits against CDS			Contig size > Average CDS size
Species	#Genes	#Contigs	#Genes		#Contigs	#Contigs
***Brachypodium distachyon***	2,043	1,797	23		113	-
***Oryza sativa***	3,281	2,791	53		351	-
***Panicum virgatum***	5,599	4,114	131		645	-
***Sorghum bicolor***	7,348	6,515	3,391		22,916	-
***Setaria italica***	3,834	3,393	74		478	-
***Zea mays***	7,809	5,656	355		1,881	-
**Total**	29,914	9,960	4,027		26,384	-
**Total unique**	29,914	9,410	3,952		26,384	4,243
**Analyses I+II+III**						
**Total grass genes**				33,941		
**Total sugarcane contigs**				40,587		
**Total unique full-length sugarcane transcripts**				9,410+3,952+4,201 = **17,563**		
**Total unique full-length sugarcane transcription factors (TFs)**				**937**		

Sugarcane transcripts were first mapped against full-length grass transcripts and were then assigned as full-length if the CDS coverage was ≥95% and the identity was ≥80% (Analysis I). In this analysis, we found 9,960 full-length contigs, which aligned against 29,914 grass genes. Analysis II took into account more than one contig that mapped to the same grass gene (Figure S2), and non-overlapped coverage was calculated. Those that fit in the criteria of coverage ≥95% and identity ≥80% were considered full-length. This analysis yielded 26,384 contigs representing 3,952 unique grass genes and therefore 3,952 full-length sugarcane transcripts. For Analysis III, we calculated the average CDS size based on six grasses (Table S2), and all contigs with a predicted protein larger than this average CDS size were considered to be full-length.

**Figure 9:**
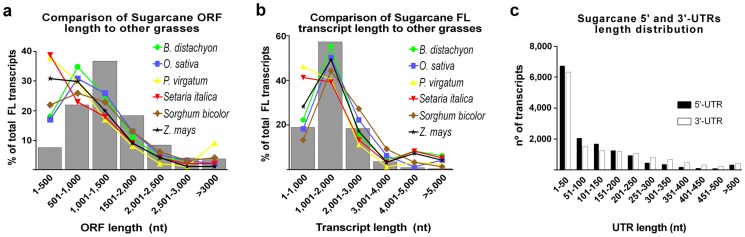
Length distribution of sugarcane ORFs, full-length transcripts (FL), and UTRs. Graphs denote the comparison of sugarcane length distribution (gray bars) of ORFs (A) and full-length transcripts (B) to other grasses (colored lines). Length distribution of 5′ and 3′ UTRs (C, black and white bars, respectively) of sugarcane full-length transcripts is shown as well.

The length of both sugarcane ORFs and transcripts follows the length distribution of other grasses full-length genes, especially when considered lengths over 1,500 nt for ORFs (Figure 9A) and 2,000 nt for transcripts (Figure 9B). Almost 40% of sugarcane ORFs presents length ranging from 1,000 to 1,500 nt (Figure 9A), whereas close to 60% of sugarcane full-length transcripts has 1,000–2,000 nt in length (Figure 9B). Besides our analysis for full-length identification primarily considered only CDS coverage as mentioned above, we can see that sugarcane full-length transcripts possess 5′ and 3′ UTRs (Figure 9C), which reinforces that our transcripts are indeed full-length sequences or, at least, comprises the entire CDS plus part of the UTRs.

A recent work with NATs in the *Arabidopsis* transcriptome showed that NATs are widespread throughout the genome and that 60% of all sense-antisense pairs overlap each other entirely [62]. Therefore, we further analyzed our dataset to identify, using the full-length analysis I and III, “full-length” NATs in sugarcane. However, only 46 (0.16% of contigs showing NATs) full-length sugarcane transcripts were fully overlapped by their antisense counterpart (Table S6). This difference in the number of NATs (28,844) and full-length NATs (46) should be interpreted carefully, since our methodology enriched for polyadenylated RNAs and there is evidence that NATs tend to be non-polyadenylated, at least in mammals [65], [66] and *Arabidopsis thaliana* [65]. Therefore, this result may suggest that sugarcane NATs, particularly full-length NATs, tend to be non-polyadenylated or rarely show polyA tail.

### Our sugarcane ORFeome comprised 937 unique, full-length transcription factors (TFs)

TFs are key regulators that allow plants to differentially regulate gene expression profiles in response to internal or external stimuli. Sugarcane TFs have been identified by microarray experiments as differentially expressed in several conditions, indicating that different TFs function in a wide range of traits and responses. Differentially expressed TFs are involved in tissue specificity [25], [67], sucrose content [23], and responses to hormones [27], cold [68], elevated CO_2_ [69], and drought [26]. Here, we identified 3,399 contigs belonging to 49 TF families (Table S7). Since the estimated gene models indicate that around 2,000 TFs are encoded in the sugarcane genome [41], our ORFeome identified a good portion of TF variants and alleles. The most abundant TF families were “far red-impaired response” (FAR1-like), “MYB-related”, and “DNA binding protein phosphatase” (DBP). Among the 17,563 unique full-length transcripts, we identified 937 unique TFs (Tables 5 and S6), therefore, we detected 46.8% (937 out of 2,000) of all unique sugarcane TFs as being full-length. These results are expected to facilitate the functional characterization of sugarcane TFs. Further analysis of these TFs would improve our knowledge of the relationship between TF expression and specific responses or traits of interest, particularly because we sampled *Saccharum* genotypes exhibiting a wide range of differing characteristics including stress tolerance, sugar content, yield and fiber content (*Saccharum spontaneum* and *Saccharum officinarum* are highly different for these traits).

## Conclusion

**Figure 10:**
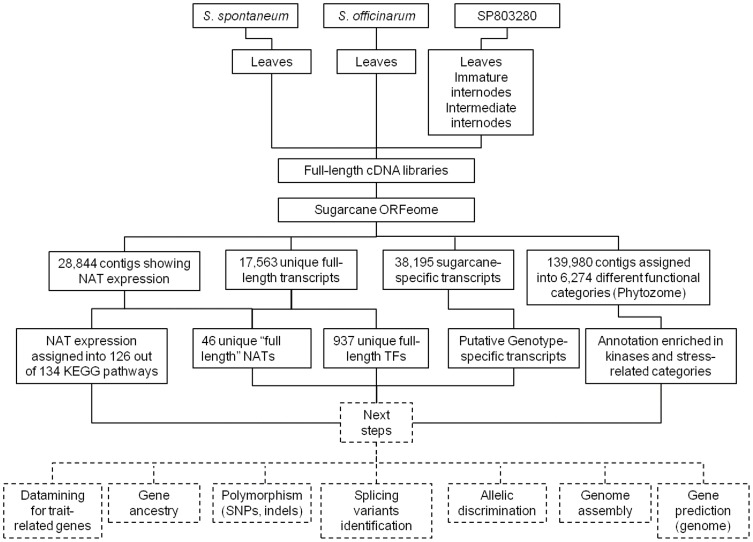
Summary of ORFeome construction and main results. The dashed line denotes future directions for sugarcane ORFeome studies. This ORFeome can be used for gene discovery related to a range of traits since over 5,000 different categories (Table S4) have a full-length representative. Differentially expressed alleles in each sample and in the hybrid and their origin from each ancestral genotype can also be analyzed. Several types of polymorphisms and genetic variability can be further investigated. Both genome assembly and annotation can make use of this sugarcane ORFeome dataset to validate and improve results. TF, transcription factor; NAT, natural antisense transcript.

In summary, we added 78,008 new transcripts to the SUCEST-FUN Database, identified 38,195 sugarcane-specific transcripts, 17,563 unique full-length transcripts (including 937 full-length TFs), putative genotype-specific transcripts, and numerous NATs affecting 126 KEGG pathways. Figure 10 summarizes our results from this initial analysis of the sugarcane ORFeome and presents the next steps that should be carried out. The use of ancestor genotypes is important for the development of the Energycane, identification of genes of interest (gene targeting), and investigation of polymorphisms and will improve our knowledge on *Saccharum* genetic variability. Furthermore, the ORFeome described in this report will provide information regarding allelic expression and ancestry, especially those related to stress responses, since the ORFeome was enriched in genes from this category. The ORFeome will facilitate assembly of the sugarcane genome since transcriptome data can be used as guides to join, order, and orient genomic fragments [70], [71]. Moreover, a high percentage of gene models (*in silico* prediction) are not experimentally verified or might be different from expressed genes [46], [72], [73]; thus, genome annotation could rely on the sugarcane ORFeome to create a better-suited matrix for gene prediction.

In conclusion, this sugarcane ORFeome represents an important advancement in sugarcane biotechnology and will be of great importance for the improvement of different areas of sugarcane research, providing a suitable reference for genomic and transcriptomic comparative analyses. This database has been made available to the scientific community at the Sequence Read Archive (SRA-NCBI) under accession number SRP042605.

## Supporting Information

Figure S1 Heat map of ‘pathway activity’ of natural antisense transcripts based on KEGG pathway annotation.
(PDF)

Figure S2 Three examples of sorghum genes covered by two or more sugarcane contigs, i.e., a full-length sugarcane gene formed by two or more contigs. Blue thick arrows denote sugarcane contigs. Lines with numbers at the top of each figure denote the position in sorghum chromosome. Gray and orange thick arrows denote UTRs and CDS part, respectively, of sorghum genes. All identities fit in the previous criteria of > = 80%. Alignments were carried out at phytozome.net against sorghum genome v2.1.Contigs with minor alignments were excluded from figure. A, alignment of two sugarcane contigs against sorghum 4-coumarate CoA:ligase gene; B, alignment of three sugarcane contigs against sorghum sucrose synthase gene; C, alignment of two sugarcane contigs against sorghum caffeic acid 3-O-methyltransferase gene.
(PDF)

Table S1 Number of Unique Contigs based on 1st Hit vs Full-Length grasses Species with Coverage >95% and Identity >80%. Species: Sb: *S. bicolor*; Zm: *Z. mays*; Pv: *P. virgatum*; Si: *S. italica*; Os: *O. sativa*; Bd: *B. distachyum*.
(XLSX)

Table S2 Number of genes, full-length genes and CDS mean size in each species from Phytozome v9.0.
(XLSX)

Table S3 Number of contigs grouped by Samples. Contigs in each genotype were analyzed by mapping the reads of each sample against the assembly (contigs). Colored lines highlight: officinarum specific contigs (green), spontaneum specific contigs (red); SP803280 specific contigs (Gray); SP803280 leaf (dark gray); ancestral specific (spontaneum + officinarum) contigs (Blue).
(XLSX)

Table S4 Annotation of sugarcane contigs in Gene ontology, KEGG, PFAM and Phytozome. Annotation of full-length contigs in Phytozome is shown separately.
(XLSX)

Table S5 Annotation of genotype-specific contigs in Phytozome.
(XLSX)

Table S6 List of “full-length” NATs and their respective annotation.
(XLSX)

Table S7 Number of transcription factors (TFs) and full-length TFs by family.
(XLSX)
