# Supplementary material for: Full-Length Enriched cDNA Libraries and ORFeome Analysis of Sugarcane Hybrid and Ancestor Genotypes
Source: PLoS One. 2014 Sep 15;9(9):e107351. doi: 10.1371/journal.pone.0107351 (PMC4164538; doi:10.1371/journal.pone.0107351)
Supplement: Figure S1 — Heat map of ‘pathway activity’ of natural antisense transcripts based on KEGG pathway annotation. (PDF) [file pone.0107351.s001.pdf]

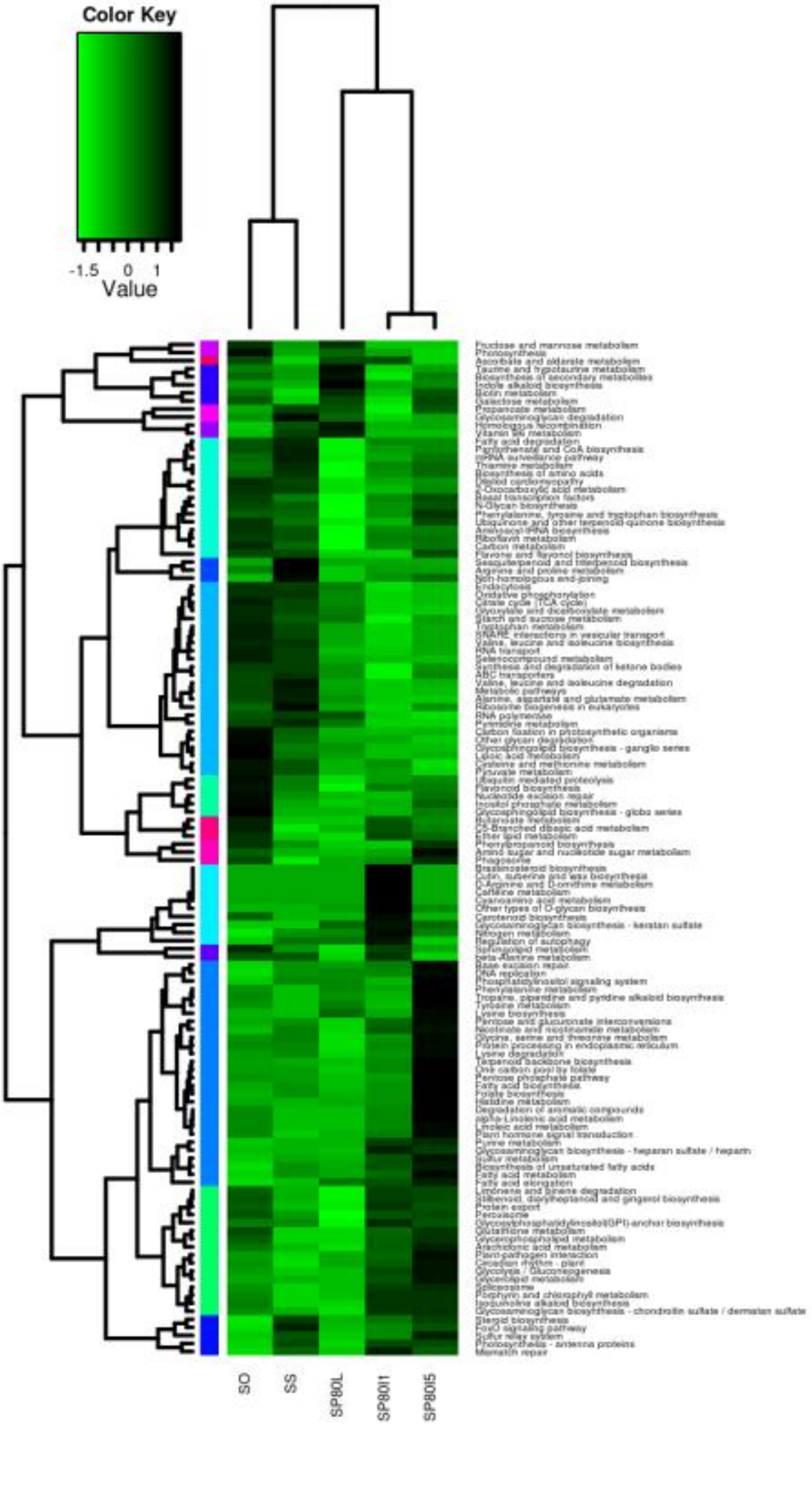

Figure S1. Heat map of 'pathway activity' of natural antisense transcripts based on KEGG pathway annotation. SO, *Saccharum officinarum* (leaf); SS, *Saccharum spontaneum* (leaf); SP80L, SP803280 (Leaf); SP80I1, SP803280 (immature internodes); SP80I5, SP803280 (intermediate internodes).
