# Supplementary material for: Full-Length Enriched cDNA Libraries and ORFeome Analysis of Sugarcane Hybrid and Ancestor Genotypes
Source: PLoS One. 2014 Sep 15;9(9):e107351. doi: 10.1371/journal.pone.0107351 (PMC4164538; doi:10.1371/journal.pone.0107351)
Supplement: Figure S2 — Three examples of sorghum genes covered by two or more sugarcane contigs, i.e., a full-length sugarcane gene formed by two or more contigs. Blue thick arrows denote sugarcane contigs. Lines with numbers at the top of each figure denote the position in sorghum chromosome. Gray and orange thick arrows denote UTRs and CDS part, respectively, of sorghum genes. All identities fit in the previous criteria of > = 80%. Alignments were carried out at phytozome.net against sorghum genome v2.1.Contigs with minor alignments were excluded from figure. A, alignment of two sugarcane contigs against sorghum 4-coumarate CoA:ligase gene; B, alignment of three sugarcane contigs against sorghum sucrose synthase gene; C, alignment of two sugarcane contigs against sorghum caffeic acid 3-O-methyltransferase gene. (PDF) [file pone.0107351.s002.pdf]

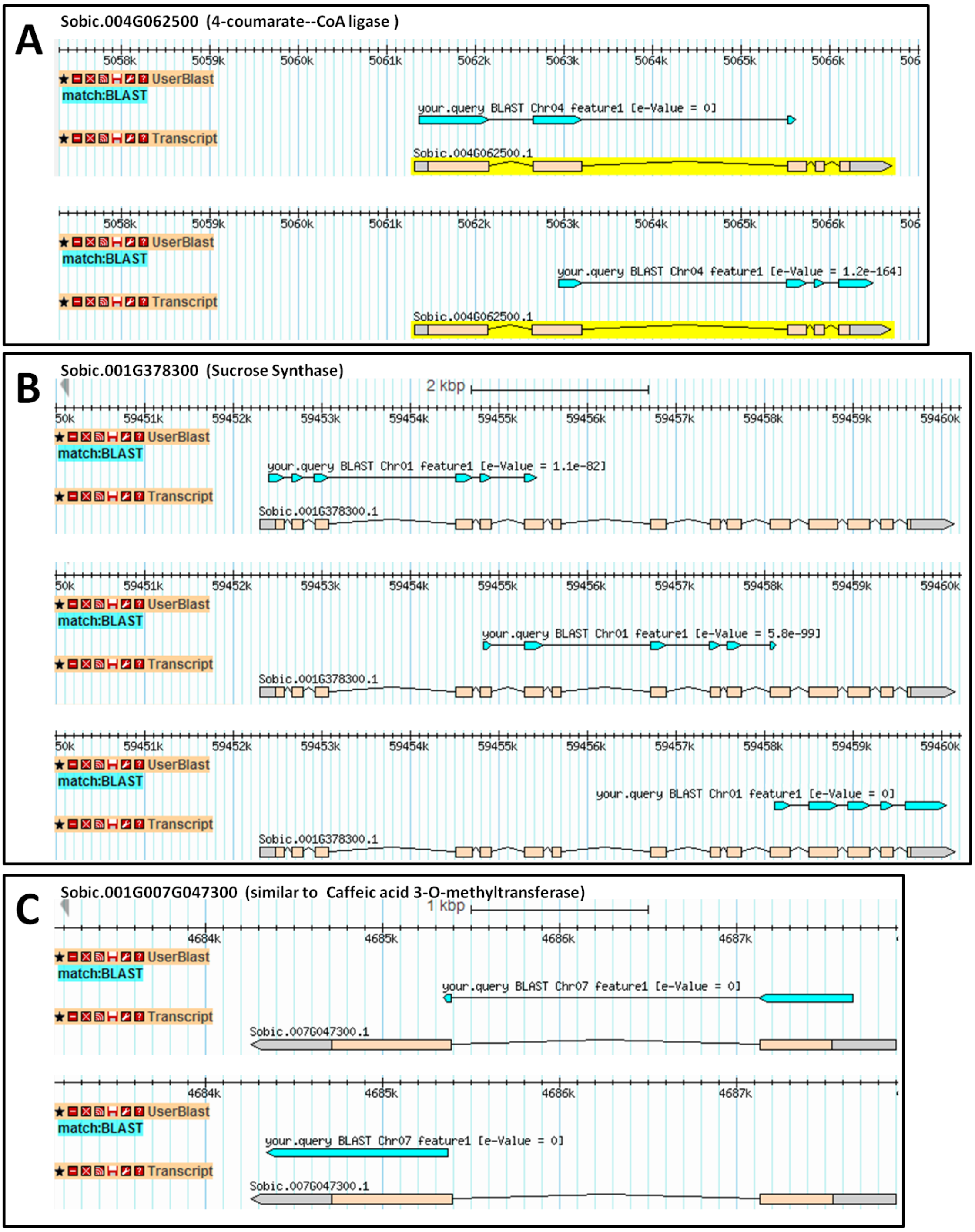

Figure S2. Three examples of sorghum genes covered by two or more contigs, i.e., a full-length sugarcane gene formed by two or more contigs. Blue thick arrows denote sugarcane contigs. Lines with numbers at the top of each figure denote the position in sorghum chromosome. Gray and orange thick arrows denote UTRs and CDS part, respectively, of sorghum genes. All identities fit in the previous criteria of >=80%. Alignments were carried out at phytozome.net against sorghum genome v2.1.Contigs with minor alignments were excluded from figure. A, alignment of two sugarcane contigs against sorghum 4-coumarate CoA:ligase gene; B, alignment of three sugarcane contigs against sorghum sucrose synthase gene; C, alignment of two sugarcane contigs against sorghum caffeic acid 3-O-methyltransferase gene.
